# Evolution of Bow-Tie Architectures in Biology

**DOI:** 10.1371/journal.pcbi.1004055

**Published:** 2015-03-23

**Authors:** Tamar Friedlander, Avraham E. Mayo, Tsvi Tlusty, Uri Alon

**Affiliations:** 1 Department of Molecular Cell Biology, Weizmann Institute of Science, Rehovot, Israel; 2 Institute of Science and Technology Austria, Klosterneuburg, Austria; 3 Simons Center for Systems Biology, Institute for Advanced Study, Princeton, New Jersey, United States of America; University of Chicago, UNITED STATES

## Abstract

Bow-tie or hourglass structure is a common architectural feature found in many biological systems. A bow-tie in a multi-layered structure occurs when intermediate layers have much fewer components than the input and output layers. Examples include metabolism where a handful of building blocks mediate between multiple input nutrients and multiple output biomass components, and signaling networks where information from numerous receptor types passes through a small set of signaling pathways to regulate multiple output genes. Little is known, however, about how bow-tie architectures evolve. Here, we address the evolution of bow-tie architectures using simulations of multi-layered systems evolving to fulfill a given input-output goal. We find that bow-ties spontaneously evolve when the information in the evolutionary goal can be compressed. Mathematically speaking, bow-ties evolve when the rank of the input-output matrix describing the evolutionary goal is deficient. The maximal compression possible (the rank of the goal) determines the size of the narrowest part of the network—that is the bow-tie. A further requirement is that a process is active to reduce the number of links in the network, such as product-rule mutations, otherwise a non-bow-tie solution is found in the evolutionary simulations. This offers a mechanism to understand a common architectural principle of biological systems, and a way to quantitate the effective rank of the goals under which they evolved.

## Author Summary

Many biological systems show bow-tie (also called hourglass) architecture. A bow-tie means that a large number of inputs are converted to a small number of intermediates, which then fan out to generate a large number of outputs. For example, cells use a wide variety of nutrients; process them into 12 metabolic precursors, which are then used to make all of the cells biomass. Similar principles exist in biological signaling and in the information processing in the visual system. Despite the ubiquity of bow-tie structures in biology, there is no explanation of how they evolved. Here, we find that bow-ties spontaneously evolve when the information in the evolutionary goal they evolved to satisfy can be compressed. Mathematically, this means that the matrix representing the goal has deficient rank. The maximal compression possible determines the width of the bow-tie—the narrowest part in the network (equal to the rank of the goal matrix). This offers a mechanism to understand a common architectural principle of biological systems, and a way to quantitate the rank of the goals under which they evolved.

## Introduction

**Fig 1:**
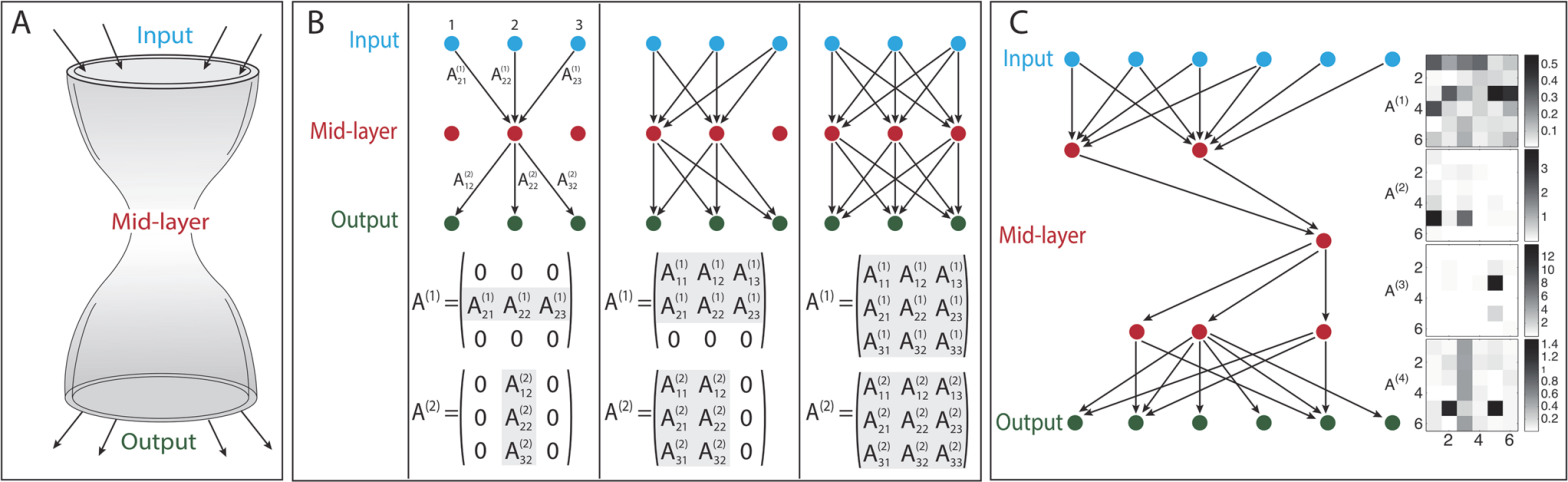
Model description. **(A)** Bow-tie in a multi-layered network means that the network is capable of processing many different inputs, by converting them into a small set of universal building blocks and then re-using these building blocks to construct a wide range of outputs. **(B) Multi-layered networks are represented by interaction intensities between components:** Our model represents a multi-layered information transmission network, by the values of interaction intensities between nodes in consecutive layers. In this schematic figure we illustrate networks with 3 layers of nodes, connected by *L* = 2 layers of interactions. It is convenient to recapitulate these interactions by *L* = 2 matrices, where the *A*_*ij*_ term in the *l*-th matrix represents the interaction between the *j*-th component in node layer *l* to the *i*-th component in node layer *l* + 1. Node layer 1 is the input signal, and node layer *L* + 1 is the output. In general, every node could be connected to every node in the next layer—as in the rightmost scheme. A bow-tie is a situation in which in one or more of the middle layers some nodes are disconnected from the rest of the network. This forms a narrow layer, termed “waist”—as exemplified in the left and middle schemes. A bow-tie architecture is captured by interaction matrices in which some rows/ columns are zero. The number of non-zero rows/ columns corresponds to the width of the waist layer. **(C) An example of bow-tie networks (simulation results):** An example of simulation results with *L* = 4 interaction layers (5 node layers), demonstrating a bow-tie of width 1 at the middle layer. The network structure is shown on the left (only active nodes shown) and the interaction intensities are shown on the right using a color code (white—no interaction, black—strong interaction).

Many natural and engineered systems show a bow-tie architecture [1,2]. A bow-tie (also termed hourglass) architecture is a feature of multi-layered networks in which the intermediate layer has significantly fewer components than the input and output layers. The intermediate layer is called the “waist” [3], “knot” [1] or “core” [4] of the bow-tie and in gene-regulatory networks the ‘input-output’ [5] or ‘selector’ gene [6]. Bow-ties mean that the network is capable of processing a variety of inputs, converting them into a small set of universal intermediates and then reusing these intermediates to construct a wide range of outputs (see Fig. 1).

A bow-tie architecture is found for example in metabolic networks [1,7–9], where the large range of nutrients consumed by the organism is decomposed into 12 universal precursors (including pyruvate, G6P, F6P, PEP, AKG, ACCOA [1,10]) from which the organism builds again all of its biomass including carbohydrates, nucleic acids and proteins. In mammalian signal transduction, a set of less than 10 pathways mediates information transfer between hundreds of possible input signals and the resulting expression changes in thousands of genes [10–13]—the same pathways are co-opted in different cell types to connect different inputs and outputs. The human visual system consists of multiple layers of signal processing, where hundreds of millions of photoreceptors in the retina fan in to only about one million ganglion cells [14] whose axons form the optic nerve. These in turn fan out to parallel processing pathways in the visual cortex that detect pattern, color, depth and movement [15]. Many developmental gene regulatory networks have bow-tie structures in which a single intermediate gene (‘input-output’ or ‘selector’ gene) combines information from multiple patterning genes (the input layers) and then initiates a self-contained developmental program by regulating an array of output genes [5,6] that can produce a large variety of morphologies [17–20]. Studies of other biological signaling networks such as epidermal receptor signaling [21], GPCR signaling [22], signaling in both the innate [23,24] and the adaptive immune system also documented bow-tie organizations [4,25].

Objects manufactured by humans do not evolve in the biological sense, however the ongoing process of technological innovation is thought to have shared features a with biological evolution [26,27]. Many non-biological networks show bow-tie architectures as well. This includes the world wide web [28], internet protocols [3], production pipelines and some economic systems—see Table 1. Bow-ties in technology have, in a sense, evolved. For example, whereas in the past each machine had its own energy source (river for mill, fire for stove), in today’s power grid a universal intermediate—220V 50Hz AC electricity—connects multiple input energy sources (coal, oil, solar etc.) to multiple output appliances [1].

**Table 1:** examples for networks having bow-tie (hourglass) architecture.

network	Input	intermediate	output	Refs.
Metabolic network	Nutrients	12 precursor metabolites (among them G6P, F6P, PEP, PYR, AKG, ACCOA)	Complex macromolecules (proteins, fatty acids, carbohydrates)	[1,7]
Developmental gene regulatory network	Patterning genes (e.g. Hox genes, *wingless*, *EGF-R*, *hedgehog*, *Notch*)	‘input-output’ or ‘selector’ gene, e.g. *shavenbaby* or *scute* in Drosophila	Developmental program of epidermis formation.	[5]
Innate immune response	>1000 microbial molecules	10 Toll-like receptors (TLRs), 4 TIR (Toll/ interleukin-1 receptor homologous region) adaptors and 2 protein kinases [24] MyD88 gene [23]	Genes responsive to NF-kB and STAT1 (>500), secondary and tertiary events (>1000)	[23,24]
Immune system	Environmental stimuli (bacteria, viruses, toxins)	Immature dendritic cells, naïve CD4+ T-cells	Various cytokines and antibody releases (B-cells, CD8+ T-cells, macrophages, etc.)	[4,25]
Signaling networks	receptors	cAMP, calcium	Genes	[21,22]
Human visual system	10^8^ photoreceptors in the eye	Optic nerve (axons of 10^6^ ganglion cells)	Image features: color, pattern, depth, movement, etc.	[14,15]
Multi-layered (deep) neural network used for clustering and dimensionality reduction	Real data is fed to upper layers (“encoder”).	“bottleneck”	Reconstructed data which is the product of the lower layers (“decoder”).	[75]
Human language writing	Typically 10^4^–10^5^ words, infinitely many numbers.	20–30 letters alphabet, 10 digits	Writing system	
Internet protocols	Physical layer (coaxial cable, optic fiber, CDMA, TDMA)	Network layer (IPv4/IPv6 protocol)	Application layer (Thunderbird, Firefox, skype)	[3]

Bow-ties have been suggested to have functional implications. Bow-ties allow evolvability, because new inputs can be readily converted to new outputs, using the same well-tested intermediate processes [2]. On the other hand, bow-ties are vulnerable to damage in the intermediate processes [1,25]. In developmental gene regulatory networks, modulated expression of the ‘waist’ (‘input-output’ or ‘selector’) gene can result in markedly different phenotypes. Thus it is thought that these ‘waist’ genes are hotspots for the evolution of novel phenotypes [5,6]. Once a bow-tie is established, it is hard to change its core components because changes to the bow-tie affect many processes at once [2,3]. Recently, Polouliakh et al. hypothesized that the narrow intermediate layer in signaling networks may serve to distinguish between different sets of inputs and assigns the correct set of outputs for each. Since this intermediate layer is narrow compared to the number of inputs, different inputs are grouped together and share a common output response [22].

The prevalence of bow-tie architectures in biology raises the question of how they evolved. In particular, one may ask whether there are evolutionary mechanisms that spontaneously give rise to bow-ties. This question is significant when considering the fact that most evolutionary simulations of multi-layered networks do not automatically give rise to bow-ties [3,29]. Generically, in fields as diverse as artificial neural networks [30] and evolution of biological networks, simulations result in highly connected networks with no bow-tie [31–37].

Bow-tie evolution in the context of internet protocol networks was studied by Akhshabi and Dovrolis [3]. Their model assumed that the node connectivity monotonously decreases between layers, namely protocols at the input layer are general in terms of their function (have many connections each), and become more and more specific towards the output layer (in which they often have only a single connection). Bow-tie structures are then a direct outcome of this inhomogeneity in properties between layers. Such assumptions are relevant for technological applications, but are not relevant in the biological context. We thus sought a biologically plausible mechanism.

We were inspired by recent advances in understanding the evolution of a different feature that is common to various biological networks—modularity. Using simulations, several studies showed that evolution under modular goals with rules that tend to eliminate connections spontaneously lead to modular structure [29,31,38–40]. This led us to ask whether one can find situations in which evolution spontaneously leads to bow-tie architectures.

Here, we study the evolution of bow-tie architecture using several simple models of multi-layered networks and biologically plausible evolutionary scenarios. We find that bow-ties evolve when two conditions are met: (i) the evolutionary goal has deficient rank; (ii) The effects of mutations on interaction intensities between components are described by a product rule—namely the mutated element is multiplied by a random number. Product-rule mutations are more biologically realistic than the commonly used sum-rule mutations which add (rather than multiply) a random number to the mutated element [41–47]. For a detailed discussion of product-mutations, their biological relevance and their evolutionary effect, the reader is referred to an earlier work [29]. We further show that the narrowest possible waist in the bow-tie is equal to the rank of the goal. We demonstrate this in simulations of evolution in linear and nonlinear model systems.

## Results

### Simulations of multi-layered network models evolving towards input-output goals

We begin with a simple linear model of a multi-layered network and later extend this framework to nonlinear models as well. The network in our model is a system that receives an input vector $\vec{s}={\vec{u}}_{1}$, performs *L* consecutive stages of processing; each stage produces an intermediate ${\vec{u}}_{2}$, ${\vec{u}}_{3}$,… ${\vec{u}}_{L}$ where the product of the final processing layer is the network output vector ${\vec{u}}_{L+1}=\vec{v}$. Here, we assume each of these processing layers can be described by a linear transformation **A**_1_, **A**_2_,…**A**_*L*_, such that the system output is $A_{L}\cdots A_{2}A_{1}\vec{s}=\vec{v}$. Each matrix contains all possible interaction intensities between network nodes at two consecutive layers. For example the matrix entry *A* ^(*l*)^_*ij*_ represents the effect of the *j*-th node in layer *l* on the *i*-th node in layer *l* + 1. In this model, connections are only possible between a node and any node at the next layer. Connections within layers or backward connections are not allowed. For illustration see Fig. 1.

As a concrete biological example, one may think of a metabolic network. The input vector is the number of nutrient molecules of different types that are consumed by the organism. Taking the example of carbohydrate metabolism: elements of the input vector $\vec{s}={\vec{u}}_{1}$ represent the amounts of the various sugars consumed: *s*_1_ could be number of glucose molecules consumed, *s*_2_ number of lactose, etc. Sugar metabolism applies a series of enzymatic reactions which first break complex sugars into simpler ones (monosaccharides) and then converts them to either of several possible output products: ATP (energy source for short term usage), glycogen (carbohydrate storage) or other sugars, for example 5-carbon sugars (pentose) used for the synthesis of nucleotides, nucleic acids and aromatic amino acids. Intermediate nodes in the model represent intermediate sugar metabolism products, such as glucose 6-phosphate, fructose 6-phosphate, pyruvate etc. [10]. The output vector represents the numbers of molecules produced using the consumed carbohydrates: *v*_1_ could be number of ATP molecules, *v*_2_ number of glycogen molecules, *v*_3_ number of ribose-5-phosphate, etc. This is admittedly a simplified description of sugar metabolism. For example, it does not take into account the hierarchical order of different sugars uptake, or metabolic cycles. Yet, it captures the degeneracy which enables replacing one sugar by another and still obtaining the very same output products. Related models have been useful for understanding multi-layered biological networks in diverse contexts, such as metabolic, gene regulatory and signal transduction networks [33,38,46,48–57].

In the linear model, the total input-output relationship of the network is given by the product of the matrices **A**_1_, **A**_2_,… **A**_*L*_, which represents the overall transfer of signals from the first (input) to the last (output) layer. Employing this formalism, we evolve these networks to match a desired goal—given by a matrix **G**. The goal matrix describes the desired output for any possible vector of inputs, and thus defines the entire input-output function. The dimension of the goal matrix **G** corresponds to the number of system inputs and outputs *D*_output_ × *D*_input_. We also extended this model to describe a gradually growing network for which the goal dimensions also change (see S1 Text).

The fitness of a network equals the distance between the total effect of the network (the product of the matrices) and the desired goal **G**, namely *F* = − || **A** ^(*L*)^ **A** ^(*L*−1)^ … **A** ^(1)^ – **G** ||. Note that each goal can be satisfied by an infinite number of matrix combinations that are all equally fit. For example, if **G** is the unit matrix **G = I** and *L* = 2, all pairs of matrices that are inverse to each other **A** ^(2)^ = [**A** ^(1)^]^−1^ satisfy the goal, because **A** ^(2)^ × **A** ^(1)^ = **I**.

To evolve the networks, we use a standard evolutionary simulation framework [37,58–60]. Briefly, the simulation starts with a population of *N* networks, each described by a set of *L* matrices. At each generation the networks are duplicated and mutated with some probability resulting in modified interaction intensities. A mutation means a change to an element of one of the matrices. Fitness is then evaluated for each structure in comparison to a goal. *N* individuals are then selected to form the next generation of the population, such that fitter individuals are more likely to be selected. This process is repeated, until high fitness evolves (see Methods section for more details).

Guided by studies on the evolution of modularity, we tested evolutionary situations which are biased to reduce or eliminate interactions. Such a mechanism is a product-rule mutation scheme in which elements of the matrices are multiplied by a random number drawn from a normal distribution N(1,σ) (as opposed to a sum-rule mutation where a random number is added instead of multiplied) [29]. Product-rule mutations are a more realistic description of the way that DNA mutations affect biochemical parameters than sum-rule mutations [41–47]. Biological mutations are more likely to decrease existing interactions than to create novel ones that did not exist before [61–63]. This property is captured by product-rule mutations (but not by sum-rule) [29]. With this mechanism, evolution finds networks satisfying the goal which are highly sparse—that is, networks with a small number of significant interactions [29,46]. As controls, we also simulated evolution with sum-rule mutations (in which a random number is added to matrix elements).

### Bow-tie architectures evolve when the goal is rank deficient

In the example of carbohydrate metabolism, different input sugars (glucose, lactose etc.) can be used to produce any of the final products (ATP, glycogen, ribose-6P). If one only examines the output molecules, one cannot tell which original sugar was their source. This degeneracy can be mathematically represented as a goal matrix with linearly dependent rows. To study the effect of these dependencies on the network structures that can evolve, we tested evolution towards goals described by matrices with different ranks. The rank *r* is the number of linearly independent rows in the matrix. The rank of the goal matrix is full, if all rows of the matrix are independent. If some of the rows are dependent, the matrix has deficient rank—a rank smaller than the full rank. Deficient rank means that the input-output transformation maps inputs to a limited subspace of outputs, of dimension *r*. Below, we discuss the implications of this concept also for nonlinear systems. As an example of a 3 × 3 matrix with rank *r* = 1 consider the following matrix whose two last rows are given by a constant multiplying the first row:

$$G=\left(\begin{array}{ccc} v_{1} & v_{2} & v_{3}\\ \alpha v_{1} & \alpha v_{2} & \alpha v_{3}\\ \beta v_{1} & \beta v_{2} & \beta v_{3} \end{array}\right).$$

Rank-deficient matrices can be decomposed into a product of (generally non-square) matrices $A^{\left(L\right)}{}_{D_{\text{output}}\times D_{L-1}}\times \cdots \times A^{\left(2\right)}{}_{D_{2}\times D_{1}}\times A^{\left(1\right)}{}_{D_{1}\times D_{\text{input}}}=G_{D_{\text{output}}\times D_{\text{input}}}$, whose smallest dimension equals the rank of the goal, namely min_*i*_(*D*_*i*_) = *r*. Because the rank of the goal matrix is smaller than its dimension *r* < *D*, this decomposition is equivalent to a narrow waist, whose width is equal to the goal matrix rank *r*. As a simple example consider the 3 × 3 goal above. It is decomposable into a product of a column vector by a row vector:

$$G=\left(\begin{array}{ccc} v_{1} & v_{2} & v_{3}\\ \alpha v_{1} & \alpha v_{2} & \alpha v_{3}\\ \beta v_{1} & \beta v_{2} & \beta v_{3} \end{array}\right)=\underset{A^{\left(2\right)}}{\underset{︸}{\left(\begin{array}{c} 1\\ \alpha \\ \beta \end{array}\right)}}\underset{A^{\left(1\right)}}{\underset{︸}{\left(\begin{array}{ccc} v_{1} & v_{2} & v_{3} \end{array}\right)}}.$$

This decomposition represents a 3-layer network, whose intermediate layer has only one active node—namely has a bow-tie structure (see left scheme in Fig. 1B). Importantly, however rank-deficient matrices can also be decomposed into products of full matrices. For any choice of **A** ^(1)^, one can find **A** ^(2)^ = [**A** ^(1)^]^−1^ **G**, as long as **A** ^(1)^ is invertible. In fact most random choices of **A** ^(1)^ will yield full-matrix decompositions which represent a non-bow-tie architecture (right scheme in Fig. 1B).

More generally, let $G_{D_{\text{output}}\times D_{\text{input}}}$ be a goal matrix with rank *r* = rank(**G**). Let there be a decomposition of $G_{D_{\text{output}}\times D_{\text{input}}}$ into a product of *L* matrices **G** = **A** ^(*L*)^ **A** ^(*L*−1)^…**A** ^(1)^. This *L* matrix decomposition is a representation of a network that has *L* + 1 layers of nodes, whereas each matrix represents the interaction intensities between all nodes in two adjacent layers. If and only if **G** has deficient rank, it can be decomposed into a product of matrices having dimensions smaller than the goal dimensions. This means that the matrices represent a network with intermediate layers that consist of fewer active nodes than the number of inputs and outputs to the network. Otherwise, if the matrix has full rank, each layer must have a number of active nodes which is at least as large as the rank, making a bow-tie impossible in a case of full rank. This argument follows from the fact that matrix multiplication cannot increase rank, i.e. rank(**A B**) ≤ min (rank (**A**), rank (**B**)) for any two matrices **A** and **B** [64].

The narrowest layer in the network is termed the waist [3]. The waist can be narrow because of the low rank that allows compressing the inputs down to fewer nodes, and then computing the outputs based on those nodes. While in principle such parsimonious bow-tie decompositions exist for every rank-deficient matrix, they constitute only a small fraction of all possible decompositions. Thus, the question remains whether evolutionary dynamics can find the solution with a bow-tie out of the infinite number of non-bow-tie solutions.

To address this question, we evolved networks towards rank-deficient goals, with and without the product-rule mutation scheme described above. We studied goals of dimension *D* = 6–8 consisting of *L* = 4–6 matrices, tested 4–8 different goals for each dimension, and evolved networks towards each goal in 100–3000 repeated simulations, each starting from different random initial conditions. We found that a rank-deficient goal together with product-rule mutations gave rise to networks that satisfy the goal and show bow-tie architectures. Full rank goals always led to evolved networks that satisfied the goal but had no bow-tie architecture at all, namely all layers had exactly the same number of nodes as the input and output layers. Rank-deficient goals with a different mutational scheme (sum rule mutations) could sometimes lead to architectures in which intermediate layers had fewer nodes than the input and output layers, but these were mostly not as narrow as the goal rank. When noise was introduced to the simulations (see below), the sum-mutation scheme led to full (non bow-tie) structures even under low noise levels. We conclude that a bow-tie evolves when (i) the goal has deficient rank, and (ii) mutation rule is product and not sum. Bow-tie width equals the rank of the goal.

For example, consider a network with 5 layers of nodes (*L* = 4 matrices of interaction layers), each consisting of 6 nodes (*D*_input_ = *D*_output_ = *D* = 6). We simulated their evolution towards goals of different ranks between 1 and full rank (*r* = 1–6). We repeated the simulation 700 times for each goal starting from different random matrix initial conditions. We then analyzed the number of active nodes at each layer. Since in a numerical simulation we do not obtain exact zeroes, but rather very small values, we defined active nodes as nodes who, if removed (incoming and outgoing interactions of the node set to zero) have a larger than 0.1% relative effect on fitness (see Methods).

**Fig 2:**
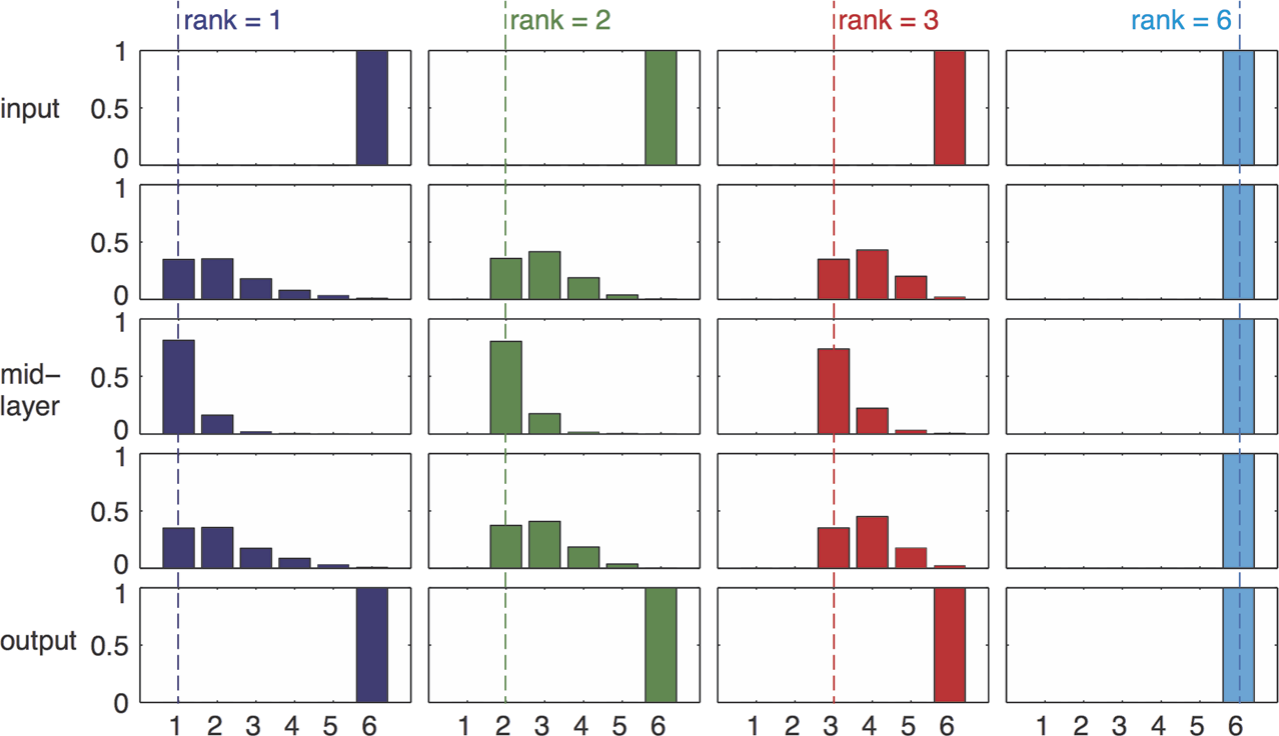
Product-rule mutations and goal which is not full rank can lead to bow-tie architecture. We show simulation results of networks with *L* = 4 (5 layers of nodes) and 6 nodes in each layer (*D* = 6). We performed 4 different sets of repeated simulations with goals of different ranks = 1,2,3 or 6. We illustrate the histograms of layer width for each set of runs. Each column in this figure shows simulation results for a different goal, and each row shows a different network layer. The number of active nodes in middle layers varies depending on the goal. The minimal number of nodes in intermediate layers (“waist”) is bounded from below by the rank of the goal. The waist width could be higher than the rank, because not all runs reach the most minimal configuration, but it cannot be lower. For example, it can be as low as 1 if the goal rank equals 1 (left column), but it is always 6 if the goal is full rank, demonstrating that no bow-tie can evolve with a full-rank goal. Simulation parameters: 3000 repeats for rank 1 and 2, 1500 repeats for rank 3 and 700 repeats for rank 6. Only runs that reached a fitness value less than 0.01 from the optimum were considered in the analysis. Product mutations were drawn from a Gaussian distribution with **σ** = 0.1, element-wise mutation rate *p* = 0.05 / *D* ^2^, tournament selection with *s* = 4.

**Fig 3:**
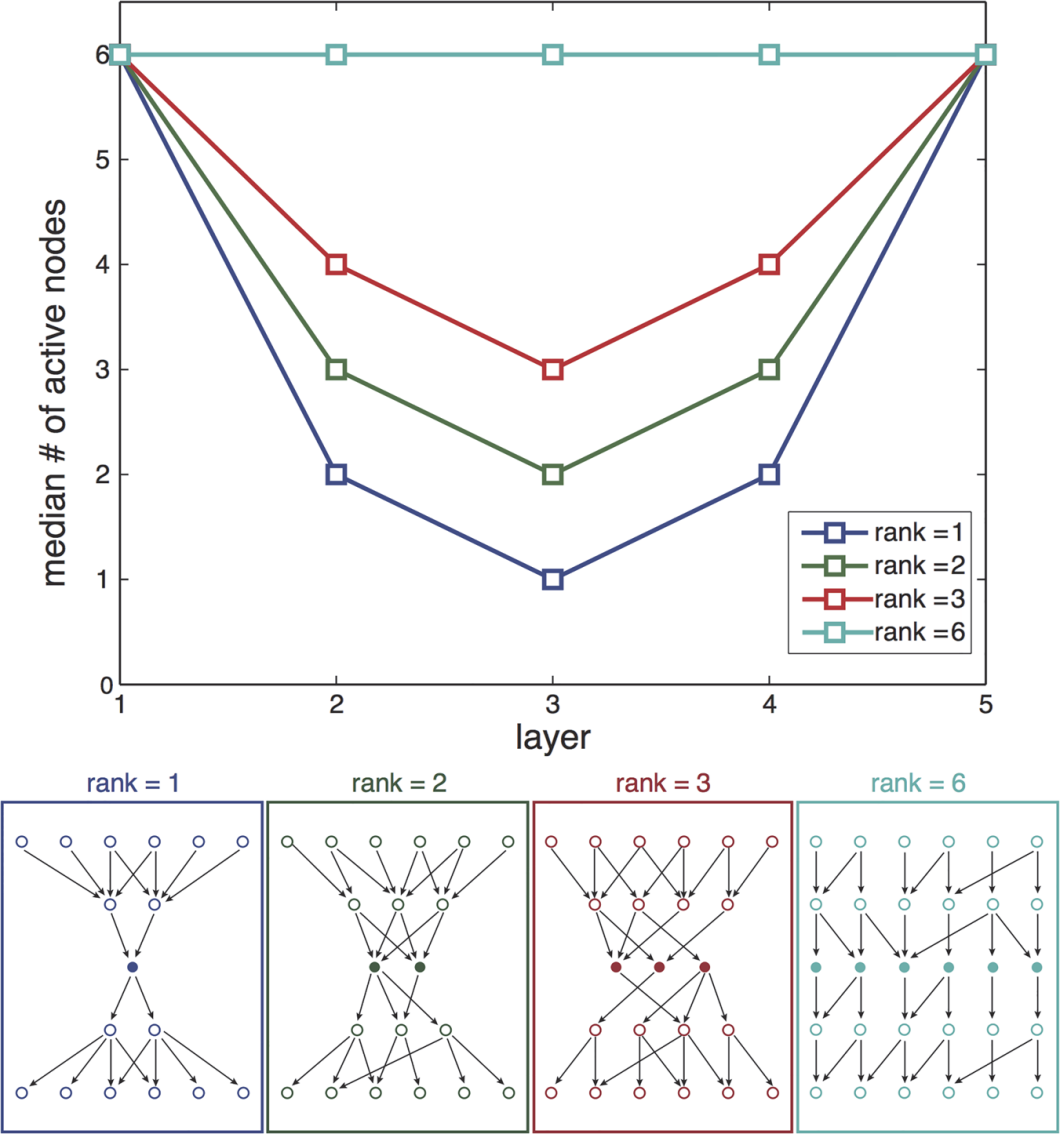
The waist is most likely to evolve in the middle layer (for equal number of inputs and outputs). **Top:** Median number of nodes at each layer. Different curves represent results for goals of different ranks. Due to symmetry considerations, the waist is most likely to evolve in the middle layer of nodes. Results refer to the same simulations as in the previous figure. Estimation of error in median calculation by bootstrapping resulted in negligible error. **Bottom:** examples of possible network structures evolved with goals having different ranks 1,2,3 and 6, illustrating how the width of waist depends on the goal rank.

We find that the number of active nodes is smallest on average at the middle layer. The number of active nodes at this waist is most often equal to the rank of the goal (Fig. 2), and never lower than this rank. The first and last layers are constrained to have exactly *D* active nodes by the definition of the problem (Fig. 3). In a minority of cases (~20%, see Table 1 in S1 Text) the waist showed more active nodes than the rank of the goal. For comparison, if the mutational mechanism is not biased to decrease interactions (i.e. sum-mutations) the vast majority (94%-97%) of the runs ended with mid-layer which had more elements than the rank of the goal (Table 1 in S1 Text). We show a representative example of a network configuration obtained in simulation in Fig. 1C.

**Fig 4:**
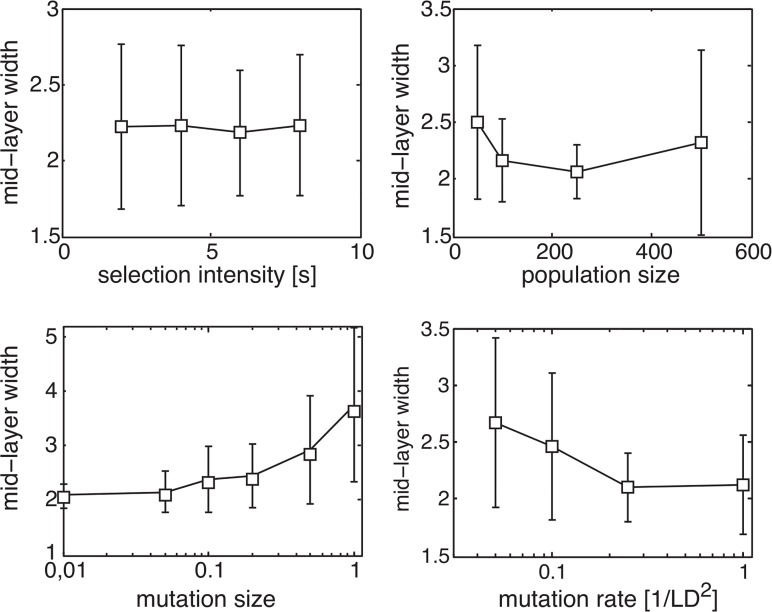
bow-tie architecture is obtained under a broad range of evolutionary parameters. **A** We tested the existence and width of bow-ties under a broad range of parameter values. We illustrate here the mean and standard deviation of the bow-tie width for various values of mutation rate, mutation size, population size and selection intensity. Bow-ties were obtained in all cases. The width of the bow-tie showed little sensitivity to the parameter values. Each point is based on 50 independent repeats of the simulation. Parameter values tested: population size = [50, 100, 250, 500]; mutation size = [0.01, 0.05, 0.1, 0.2, 0.5, 1]; mutation rate = [1, 0.25, 0.1, 0.05]/*LD* ^2^, tournament size *s* = [2, 4, 6, 8].

We tested the sensitivity of this mechanism for bow-tie evolution to model parameters. A bow-tie was obtained under a wide range of values of selection intensity, mutation size, mutation rate and population size that spanned 1.3 decades (mutation rate) to 2 decades (mutation size) (Fig. 4; see S1 Text for more details). We also tested the sensitivity of the structure obtained to the evolutionary goal by comparing simulation results with different goals having the same rank. We find that the location and width of the waist are insensitive to the choice of the goal (see Fig. 9 in S1 Text).

We tested the location of the waist in simulations of multi-layered networks with equal number of inputs and outputs (*L* = 4, *D* = 6; *L* = 6, *D* = 8). While in principle the waist could reside at any layer between the input and output layers, in practice, it falls most often in the middle layer. Intuitively, this can be explained by symmetry considerations: The mutational mechanism works uniformly on all layers to eliminate connections. While the dimensions of the goal matrix constrain the number of active nodes at the network boundary layers (input and output), connections near the middle layer are least “protected” and thus mostly prone to removal, resulting in the network waist being on average in the middle layer.

### Bow-tie architecture also evolves under temporal noise

**Fig 5:**
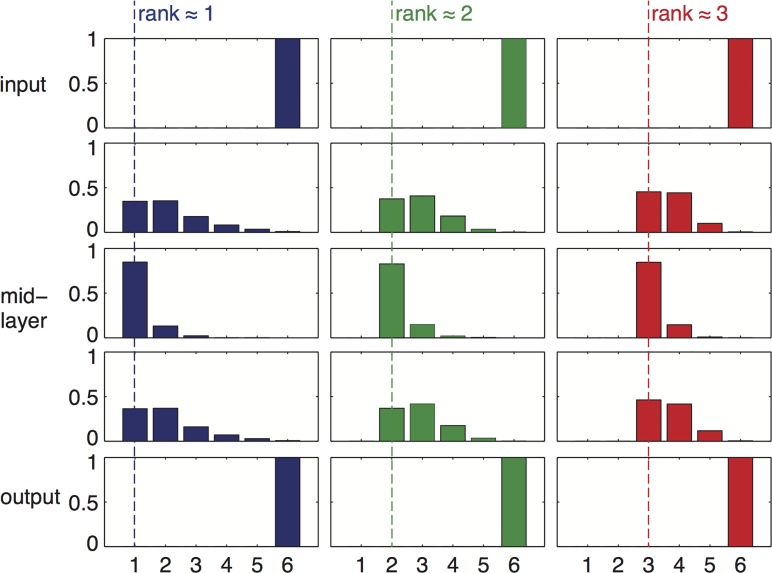
Bow-tie evolves even if the goal is only approximately of deficient rank. We show simulation results when the goal consisted of a matrix of deficient rank (1, 2 or 3) to which some level of noise was added (see Methods), so mathematically speaking goals had full rank, such that some of the eigenvalues were relatively small. Remarkably, here too a bow-tie architecture evolved, however the width of the waist was not as narrow as if the goal had exact noiseless deficient rank (compare to Fig. 2). For each goal rank we calculated layer activity statistics based on 1500 different runs (each having a different goal, but with same noise statistics). Noise level here was 1% (averaged over all runs analyzed) for all ranks. This result demonstrates that the evolutionary process can expose a deficient goal rank even when noise is added, as is expected to be the case in realistic systems. Other parameters are the same as in Fig. 2.

Biological networks are often prone to fluctuations, for example due to temporal variations in internal molecule numbers or environmental fluctuations. We thus tested the robustness of the suggested evolutionary mechanism to fluctuations in either the goal or the interaction intensities. We started by testing the sensitivity of the evolutionary mechanism to rank accuracy by perturbing the rank-deficient goal matrix, yet keeping the goal constant throughout every simulation run. This produced goal matrices that are ‘almost rank deficient’: full rank, but with some of the eigenvalues close to zero. The noise strength is given by the difference between the norms of the noisy and clean goals divided by the norm of the clean goal (see Methods). We find that for noise strength up to about 1%, bow-tie architecture with middle layers whose width equals the goal rank were reached in most simulation runs, just as in the absence of noise. Thus, our evolutionary simulation is robust to small perturbations to exactly rank-deficient goals—see Fig. 5 for illustration and compare to Fig. 2 with no noise. The median waist size increased above the clean rank when noise intensity increased above 1% (see Figs. 14–15 in S1 Text for the dependence of bow-tie on the noise level). For estimation of the noise magnitude in biological networks, see S1 Text.

While in the previous scenario the goal rank was noisy, but remained constant throughout every simulation run, temporal fluctuations are also ubiquitous in biological networks. To test the effect of temporal fluctuations, we added statistically independent noise realizations (white noise) to all matrix entries (and also to the goal) at each generation. The fitness evaluation then reads: *F* = − || (**A** ^(*L*)^ + **ε**_*L*_)(**A** ^(*L*−1)^ + **ε**_*L*−1_)…(**A** ^(1)^ + **ε**_1_) – (**G** + **ε**_*G*_) ||, where **ε**_*i*_ are independent noise realizations. Since this noise changes at a higher frequency than the typical evolutionary timescale (the mutation rate), we expected that the system will be able to filter it out to some extent. Since here the noise affects all network components and not only the goal, we refer to the induced fluctuations in fitness as a global measure of the noise intensity. We compared the ability of evolution with either product or sum mutations to cope with this temporal noise. We find that product-mutations filter out the noise much more efficiently than sum-mutations. When the clean goal rank was 1, the network structure evolved by product-rule evolutionary scheme was unaffected until the relative magnitude of induced fluctuations in fitness reached values of 0.3 (std/mean). Sum mutations, in contrast, led to bow-tie of width 3 (compared to the minimal width in this case, 1) even in the absence of noise; bow-tie width sharply increased to 5 when temporal noise was added. Since complete absence of noise is a non-realistic scenario in biological systems, we conclude that sum-mutations cannot account for bow-tie evolution. See Fig. 16 in S1 Text for illustration.

### Bow-ties can evolve in nonlinear information transmission models

Finally, we asked whether the present mechanism would apply in a nonlinear network model. While goal rank is a straightforward measure of dimensionality in linear systems, the concept of rank is more elusive when it comes to nonlinear systems. Yet, one can intuitively think that a similar concept could exist there too. To test this hypothesis we employed a well-studied problem of image analysis using perceptron nonlinear neural networks [65,66]. In this problem, each node integrates over weighted inputs and produces an output which is passed through a non-linear transfer function, **u** ^(*l*+1)^ = *f* (**A** ^(*l*)^ **u** ^(*l*)^ – **T** ^(*l*+1)^), where **A** ^(*l*)^ and **T** ^(*l*)^ are the weight matrix and corresponding set of thresholds in the *l*-th layer, and **u** ^(*l*)^ is the set of inputs propagated from the previous layer (see Methods).

**Fig 6:**
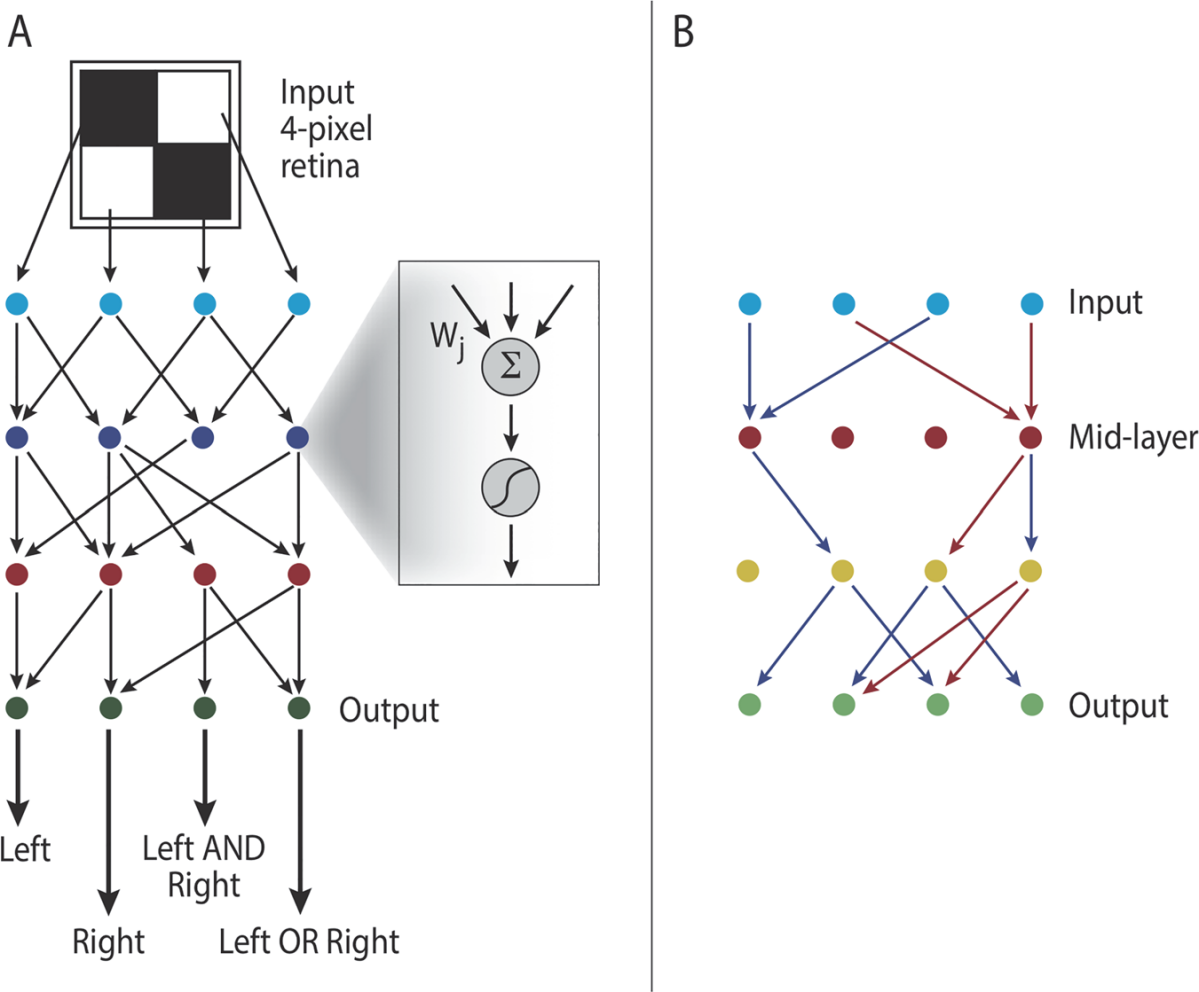
Bow-tie can evolve for a nonlinear input-output relation too, if the input can be more compactly represented with no effect on the output. We show simulation results of a simple nonlinear problem mimicking a 4-pixel retina. **(A) Problem definition**: The retina has four inputs (one for each pixel, that can be either black or white), four outputs and two internal processing layers. The retina is evolved so that its outputs detect whether there is (i) an object on the left side (at least one pixel in the left column is black), (ii) on the right side (at least one pixel in the right column is black), (iii) left AND right objects, (iv) left OR right objects, correspondingly. Inset: in contrast to previous problems, here each node performs a nonlinear transformation of the sum of weighted inputs: **u** ^(*l*+1)^ = *f* (**A** ^(*l*)^ **u** ^(*l*)^ – **T** ^(*l*+1)^), where **A** ^(*l*)^ and **T** ^(*l*)^ are the weight matrix and set of thresholds in the *l*-th layer. **(B) Typical example of simulation results**. Apparently, two bits of information are sufficient to fully describe the four required outputs in this model. Indeed, the network evolved so that it has only two active nodes in the second layer (red circles).

We evolved the networks towards a goal of identifying features in a 2 × 2 retina with Boolean pixel values (*D*_2_ = 4 inputs) (Fig. 6). Low dimensionality was achieved by defining as a goal four outputs that depend only on two features of the image. The four required Boolean outputs were: (a) at least one pixel in the left retina column, (b) at least one pixel in the right column, (c) pixels in both left and right columns, (d) pixel(s) in the left or in the right columns. These four outputs can be fully represented by only two features: (a) and (b), making the 4-dimensional input space redundant. Thus, the effective “rank” here is *r* = 2.

Simulations evolving the weights {*A*_*ij*_^(*l*)^} and thresholds {*T*_*i*_^(*l*)^} values using product-rule mutations that led to nearly perfect solutions (fitness of less than 10^−4^ from the optimum) mostly had a narrow waist: one of the intermediate layers had only two active nodes in 75% of the runs (see Table 1 in S1 Text). For comparison, simulations with a mutation rule that was not biased to eliminate interactions (sum-rule) were much less likely to lead to networks with a narrow waist (this was observed in only 45% of runs). Detailed statistics over 500 runs of network structures obtained with either mutational scheme is presented in Fig. 12 in S1 Text.

## Discussion

We studied the evolution of bow-ties in layered networks. We find that bow-ties evolve spontaneously when two conditions are met: the goal has deficient rank and the effect of mutations on interactions is well-approximated by a product-rule. The size of the narrowest layer—the waist of the bow-tie—is bounded from below by the rank of the goal. We find the evolution of narrow waists in a wide range of evolutionary parameters, in both linear and nonlinear multi-layered network models. We find that bow-tie structures can also evolve under temporal noise, if the mutational scheme is approximated by a product-rule. An alternative mutational scheme—sum-rule—proved much more vulnerable to noise and did not lead to bow-tie structures.

The concept of rank is defined clearly in the case of matrix-like goals and linear transfer functions. In more complex situations, such as the nonlinear retina problem and gene regulatory networks, the rank corresponds to the minimal number of input features on which the outputs depend. One may hypothesize that in the case of probabilistic time dependent signaling in cells and nervous systems, rank may be related to the information theory measure of information source entropy. This is the minimal number of bits which is sufficient to encode the source [67]. A natural information source (“input”)—such as biological signals—is often redundant. Its compression (source coding) can shorten the description length while still preserving all the necessary information (“waist”). In analogy to the goal rank, the shortest possible description equals the source entropy. The present results can supply an operational definition of the goal rank in a layered nonlinear system—the minimal evolved waist under the present assumptions [68–74].

Here we considered the input-output relation as the sole force guiding the evolution of the network, however there may be other constraints or processes affecting network structures. For example, in the visual system, the fan-in of ganglion cells into the optic nerve was suggested to be partially due to space limitation [16]. A recent study, suggested that bow-ties in developmental gene regulatory network can evolve due to hierarchy in specificity [79]. Cross-talk between networks as well as addition and deletion of network nodes can also influence network structure. A previous study [53] focused on the contribution of robustness to evolving network topologies. It suggested that connectivity can vary between genes in a network, such that genes that buffer genetic variation are highly connected, although the overall network is sparse.

Bow-tie structures are also common in multi-layered artificial neural networks used for classification and dimensionality reduction problems. While there are parallels in the functional role of bow-ties there with the biological bow-ties which are the focus of this study, these artificial neural networks are designed a priori to have this bow-tie structure. Multi-layered neural networks often use an intermediate (hidden) layer whose number of nodes is smaller than the number of input and output nodes [30,75]. There, the role of the hidden layer is to capture the significant features of the inputs. The favorable usage of bow-tie structures in neural networks suggests that often the number of important features is lower than the number of inputs [65]. The transformation between input and hidden layer was shown to map the data into a space in which discrimination is easier [76,77].

A similar functional principle was observed in several signaling networks—where a large number of input signals funnel through a narrow intermediate layer to produce a limited number of output programs [22,23,78]. Kitano and colleagues [22,23] highlighted the structural similarity between these biological bow-tie networks and neural network classifiers.

Previous studies debated over the role of noise in shaping complex network architecture. Some argue that there is a trade-off between compression and noise mitigation relying on information theory arguments [73,80]; others suggest that thermal noise can aid funneling evolutionary dynamics, and avoiding local extrema when the fitness landscape is rugged [81]. The evolutionary process in our model can filter out temporal noise to some extent and still produce bow-tie structure manifested by an intermediate layer whose number of active nodes equals the goal rank. This results from the separation of timescales between the evolutionary process which is driven by the mutation rate (slow) and the temporal noise (fast). The evolutionary process can then average out the rapid temporal fluctuations. Evolution under product-mutations characteristically has dynamical attractor states (such as zero rate constants, which remain zero upon multiplication by a number), in contrast to evolution under sum-mutations [29]. This dynamical stability renders the product-mutation landscape more noise-proof than the sum-mutation one. These results call for further research to better understand the multiple roles played by noise in the evolution of complex networks.

When a biological network expands gradually such that the goal rank remains intact, the bow-tie is usually “ossified” (see S1 Text and Fig. 17 in S1 Text). Namely, a bow-tie node(s) established when the network is small is very likely to remain a bow-tie rather than being replaced by another node. Thus bow-tie nodes end up as among the most ancient in the network. This induces a correlation between node connectivity and its evolutionary age. It would be interesting to validate this prediction by testing whether bow-tie network elements are indeed the most ancient ones.

One may speculate whether the relation of bow-tie width to goal rank may be instructive in fields outside of biology. As an example of compression by bow ties, consider alphabets. The entire vocabulary of a language can be transmitted between people using a bow-tie of 20–30 characters. This is not the only possible design: syllabaries such as Japanese Kana represent syllables instead of the vowels/consonants of alphabets, and logographies such as Chinese represent words. The size of the bow-tie in each case may be hypothesized to be close to the minimum required for capturing each level: many tens of syllables, and many thousands of words. Efficiency considerations are probably at play as a ‘selective force’: comparing number systems such as Arabic numerals to Roman numerals shows a progression from a cumbersome to a more efficient bow-tie description.

Taken together, our results suggest a mechanism for the evolution of bow-tie architectures in biology and a way to quantitate the rank of the evolutionary goals under which they evolved.

## Methods

### Evolutionary simulation

The evolutionary simulation was written in Matlab using standard framework [58–60]. The source codes and analysis scripts are available as supporting materials. We initialized the population of matrices by drawing their *N* ⋅ *LD* ^2^ terms from a uniform distribution. Population size was set to *N* = 100. Each “individual” consists of a set of *L* matrices. In each generation the population was duplicated. One of the copies was kept intact, and elements of the other copy had a probability *p* to be mutated—as we explain below. Fitness of each of the 2*N* individuals was evaluated by *F =* − || **A** ^(*L*)^ **A** ^(*L* – 1)^ … **A** ^(1)^ – **G** ||, where || ⋅ || denotes the sum of squares of elements [82]. The best possible fitness is zero, achieved if **A** ^(*L*)^ **A** ^(*L* − 1)^ …**A** ^(1)^ = **G** exactly. Otherwise, fitness values are negative. We constructed the goal matrices from combinations of ‘0’ and ‘10’ terms. We tested goals of different ranks and different internal structures and found no sensitivity for goal details other than its rank (see S1 Text). *N* individuals are selected out of the 2*N* population of original and mutated ones, based on their fitness (see below). This mutation–selection process was repeated until the simulation stopping condition was satisfied (either a preset number of generations or when mean population fitness was within 0.01 of the optimum).

#### Mutation

We mutated individual elements in the matrix. We set mutation rate such that on average 20% of the population members were mutated at each generation, so the probability of each matrix element to be mutated was $∼\frac{0.2}{LD^{2}}.$ This relatively low mutation rate enables beneficial mutants to reproduce on average at least 5 generations before an additional mutation occurs. We randomly picked the matrix elements to be mutated. Mutation values were drawn from a Gaussian distribution (unless otherwise stated). The mutated matrix element was then multiplied by the random number: *A*_*ij*_^(*l*)^ → *A*_*ij*_^(*l*)^ ⋅ N(1,σ). In simulations we used σ in the range 0.01–1. Maximal achievable fitness and the timescale to convergence depend on the mutation frequency and size, as demonstrated in our sensitivity test (see S1 Text).

#### Selection methods

We used tournament selection with group size *s* = 4 (see [60] chap. 9). In a previous work we tested 2 other selection methods (truncation-selection (elite) [58] and proportionate reproduction with Boltzmann-like scaling [46,55,83]) and found that all three methods gave qualitatively very similar results with only a difference in time scales.

#### Noisy goals

In order to test the effect of noisy rank we added a low amount of noise to the goals used in the previous simulations. We used goals with ranks 1, 2 and 3 whose terms were either 10 or 0 and then added a uniformly distributed noise in the range [0, 0.1] (σ = 0.029). We define the noise level as the absolute value of the difference between the norms of the noisy and clean goals divided by the norm of the clean goal: $\left|\frac{\left||G^{*}||-||G|\right|}{\left||G|\right|}\right|$, where **G** is the ‘clean goal’ and G* is the noisy one. As norm we took the sum of squares of all matrix terms. At every repeat of the simulation we added a different noise realization with the same statistics. The noise (and thus the evolutionary goal) was fixed throughout any given run. The noise intensity was calculated separately for each run. The values presented are averaged over all runs considered in the analysis.

#### Temporal noise

To test the effect of temporal fluctuations, we added statistically independent noise realizations (white noise) to all matrix entries (and also to the goal) at each generation. The fitness evaluation then reads: *F =* − || (**A** ^(*L*)^ + **ε**_*L*_)(**A** ^(*L* – 1)^ + **ε**_*L* – 1_) … (**A** ^(1)^ + **ε**_1_) – (**G** + **ε**_*G*_) ||, where **ε**_*i*_ are independent noise realizations drawn from a Gaussian distribution N(0,σ) with different values of σ varying between 0.001 to 0.2. We measured the overall effect of temporal fluctuations at every node by calculating the standard deviation in fitness values in the last 5000 generations of the simulation, when the run has already converged (runs were for either 50,000 or 100,000 generations each).

### Data analysis

Repeated simulations were run using the same parameters, where at every single run the Matlab random seed was initialized to a different value. Consequently, each run starts from different initial conditions and uses different mutational realizations. In the analysis, we checked whether the runs converged. Only runs that gave results within 0.01 from the optimum were considered in the analysis. We then analyzed in each run the number of active nodes in the layer (see below). In the figures we show either the median number or histogram of active nodes per layer, when applicable.

#### Active nodes

To calculate the number of active nodes in a layer, we eliminated each node at a time, by equating to zero all its input and output interactions. For example to eliminate the *k*-th node in layer *l* + 1 we set *A* ^(*l*)^_k,*_ = *A* ^(*l* + 1)^_*,*k*_ = 0, leaving all other terms intact. We then calculate the fitness value of the modified network $\tilde{F}$ and define the difference compared to the original fitness value *F*: $\Delta F=|F-\tilde{F}|$. We compare Δ*F* / *F* between all nodes located at the same layer. A node whose relative effect on fitness is less than 0.1% is considered inactive.

### Retina problem

We tested the evolution of bow-tie networks in this non-linear problem which resembles standard neural network studies [39,65,84]. We defined a problem with 4 inputs and 4 outputs and 2 internal processing layers consisting of 4 nodes each. The inputs represent a 4-pixel retina, where each pixel could be either black or white, as described in the results section.

The evolutionary simulation followed a similar procedure to the linear problem described above. Mutation, selection, and data analysis methods were similar to the ones used in the linear problem as described above. The main difference is that the output of each layer was not a linear function of the inputs as before, but rather a non-linear function **u** ^(*l* + 1)^ = *f* (**A** ^(*l*)^ **u** ^(*l*)^ – **T** ^(*l* + 1)^), where **A** ^(*l*)^ and **T** ^(*l*)^ are the weights matrix and corresponding set of thresholds in the *l*-th layer correspondingly, and **u** ^(*l*)^ is the set of inputs propagated from the previous layer. The non-linear transfer function *f* was rescaled to range between 0 and 1, *f*(*x*) = (1 + tanh(*x*)) / 2. The result of this computation is fed to the next layer until the last (output) layer is reached. In non-linear systems the evolutionary goal cannot be described by a single goal matrix as in the linear case. Rather, it is defined by pairs of input /output relations. The evolutionary simulation tested all possible inputs simultaneously, and evolved the network parameters to provide the correct output in each case. The fitness was defined as the difference between the network output and the desired output, in similarity to the linear model and then averaged over all possible input/output pairs. Inputs and outputs were encoded by Boolean vectors. Internal layer calculation used continuous values, but simulations could reach very high precision (≤ 10 ^−10^ from the optimum). Simulations were run for 10^4^ generations. Only runs that reached fitness within 10^−4^ of the optimum were considered in the analysis.

The retina simulation was written in Wolfram Mathematica. We initialized the population of matrices and corresponding thresholds by drawing their *N* ⋅ *LD*(*D* + 1) terms from a uniform distribution in the range [-2,+2]. Population size was set as *N* = 100. In each generation the population was duplicated. One of the copies was kept intact, and elements of the other copy had a per-term probability *p* = 0.2 to be mutated. The mutation was implemented through multiplying the mutated term by a random number drawn from a normal distribution with mean 1 and std 0.5 (thus a probability of about *q* = 0.02 to change sign).

To determine active nodes in this case, we begin by setting each weight to zero in its turn *A* ^(*l*)^_*ij*_ = 0 leaving all other terms intact. This procedure was not applied to the threshold values *T*_*i*_^(*l*)^ because a node may be left in the network, even if no inputs are propagated through it from an upper layer. In these cases the role of such a node is to introduce a constant bias set by its threshold. We then calculate the fitness value of the modified network $\tilde{F}$ and define the difference compared to the original fitness value: $\Delta F=|F-\tilde{F}|$. We compare Δ*F* / *F* between all weights located at the same layer. A network interaction whose relative effect on fitness is less than 10^−4^ was set to zero. A node whose entire set of outgoing weights was set to zero was considered inactive.

## Supporting Information

S1 Text Additional figures and simulation results as follows: 1.Parameter sensitivity test, 2. The emergence of bow-tie is insensitive to the internal goal structure (as long as the rank remains intact), 3. Sum-mutations are less likely to lead to narrow bow-tie structures compared to product-mutations, 4. Fraction of runs that did not converge to a bow-tie with narrow layer that equals the goal rank, 5. A bow-tie evolves even if the product-mutations can change interaction sign, 6. Bow-tie dependence on noise level added to the goal, 7. Product-mutations can filter temporal noise efficiently and lead to bow-tie; sum-mutations cannot. 8. Estimation of noise in a biological network, 9. Change in network size—bow-tie is typically ossified. (DOCX)

S1 Dataset Evolutionary simulation code. (7Z)
