# Supplementary material for: Evolution of Bow-Tie Architectures in Biology
Source: PLoS Comput Biol. 2015 Mar 23;11(3):e1004055. doi: 10.1371/journal.pcbi.1004055 (PMC4370773; doi:10.1371/journal.pcbi.1004055)
Supplement: S1 Text — (DOCX) [file pcbi.1004055.s001.docx]

**Evolution of bow-tie architectures in biology**

Tamar Friedlander, Avraham E. Mayo, Tsvi Tlusty and Uri Alon

**Supporting Information Text**

Table of Contents

[1. Parameter sensitivity test 2](#_Toc402944801)

[2. The emergence of bow-tie is insensitive to the internal goal structure (as long as the rank remains intact) 10](#_Toc402944802)

[3. Sum-mutations are less likely to lead to narrow bow-tie structures compared to product-mutations 12](#_Toc402944803)

[4. Fraction of runs that did not converge to a bow-tie with narrow layer that equals the goal rank 16](#_Toc402944804)

[5. A bow-tie evolves even if the product-mutations can change interaction sign 17](#_Toc402944805)

[6. Bow-tie dependence on noise level added to the goal 18](#_Toc402944806)

[7. Product-mutations can filter temporal noise efficiently and lead to bow-tie; sum-mutations cannot. 20](#_Toc402944807)

[8. Estimation of noise in a biological network 21](#_Toc402944808)

[9. Change in network size – bow-tie is typically ossified 23](#_Toc402944809)

# Parameter sensitivity test

Evolutionary simulations results depend on a number of key parameters. Here we tested the sensitivity of our simulation results: the emergence of bow-tie structures with narrow intermediate layer, best population fitness reached and fitness temporal trajectories to the following simulation parameters:

1. Selection intensity (size of tournament)
2. Mutation size
3. Mutation rate
4. Population size.

All parameters affect both the speed of fitness convergence and the final fitness value achieved to some extent. Bow-tie width was found insensitive to the selection intensity and weakly sensitive to population size. It showed some dependence on mutation size and rate. Yet, bow-ties did evolve under a broad range of values for all these parameters.

Parameters for all runs: $L=4,D=6$ (namely a network of 5 node layers with 6 nodes in each), goal: $\mathbf{G}=\left[\begin{matrix} \begin{matrix} 10 & 10 & 10 \\ 10 & 10 & 10 \\ 10 & 10 & 10 \end{matrix} & 0 \\ 0 & \begin{matrix} 10 & 10 & 10 \\ 10 & 10 & 10 \\ 10 & 10 & 10 \end{matrix} \end{matrix}\right]$with rank 2.

Here we detail the values tested for these parameters and the default values used otherwise.

Mutation values were drawn from a normal distribution with $\text{N}(1,\sigma)$. Default mutation size (except for mutation size runs) used was $\sigma=0.1$. In runs testing mutation size we tested the values $\sigma=$0.01, 0.05, 0.1, 0.2, 0.5, 1.

Default mutation rate (per matrix entry) used $R_{0}/5$, where $R_{0}=1/LD^{2}$. In runs testing mutation rate we tested the values: $R_{0}$, $R_{0}/4$, $R_{0}/10$, $R_{0}/20$.

Default tournament size (except for tournament size runs) $s=4$. In runs testing selection intensity we tested the values $s=$2, 4, 8. This parameter determines the selection intensity.

Default population size was 100. In runs testing population size effects we tested the values 50, 100, 250, 500.

In the following we illustrate examples of temporal fitness trajectories (population mean) and histograms of layer width with different parameter values.

**Selection intensity**

Selection intensity had a negligible effect on the bow-tie structure, and was mainly affecting the speed of convergence to better fitness (see Figs. S1, S2). The stronger is the selection (larger tournament size), the closer is the fitness to the optimum at a given number of generations. Note that the figures show the absolute value of fitness (which is the distance from the optimum) – so the curves are monotonously decreasing.

Fig. 1: The Stronger the selection, the better is the fitness achieved at a given number of generations. We show here examples of fitness temporal trajectories with different tournament size parameter (determines selection intensity). Other parameters are the same in all three simulations.

Fig. 2: The emergences of narrow bow-tie (and in general layer width) are insensitive to selection intensity. Simulation results showing layer width statistics for different selection intensity values, where all other parameters remain intact. Statistics is based on 100 different runs for each selection intensity value.

**Mutation size and rate**

Examining the effect of mutation size and rate on temporal fitness trajectories, one notices two phases: fast convergence at the first phase followed by slow convergence at the second phase. The larger is the mutation size / rate the faster is the fitness convergence in the initial phase, but it is then “stuck” at a fitness value which is further from the optimum in the second phase. This can be thought of as a “noise floor” defined by the mutations (see Figs. 3 and 5).

Generally, all values of mutation size and rate tested show a bow-tie architecture. The smaller is the mutation size (the higher the mutation rate), the closer is the width of the bow-to the minimal size possible. In this case the rank of the goal was 2, which defines the minimum – see Figs. 4, 6.

Fig. 3: Mutation size affects both the speed of fitness convergence at the initial phase and the finite fitness value reached at the second phase. The figure shows examples of fitness temporal trajectories with different values of mutation size (other parameters are the same). The smaller is the mutation size, the slower is the convergence at the first phase, but the final fitness reached is higher. Note that the red curve (mutation size = 0.01) did not converge in the time tested here.

Fig. 4: The smaller is the mutation size, the narrower is the waist layer. Simulation results showing layer width statistics for different mutation size values, where all other parameters remain intact. Statistics is based on 50 different runs for each mutation size value. Runs lasted 100,000 generations each regardless of the fitness value. Typically, fitness values reached were very close to the optimum (order of 10^-4^-10^-6^ from the optimum).

Fig. 5: Mutation rate affects both the speed of fitness convergence at the initial phase and the finite fitness value reached at the second phase. The figure shows examples of fitness temporal trajectories with different values of mutation rate, where $R_{0}=1/LD^{2}$. Other simulation parameters are the same for all curves.

Fig. 6: The higher is the mutation rate, the narrower is the waist layer (for equal number of generations). Simulation results showing layer width statistics for different mutation rate values, where all other parameters remain intact. Statistics is based on 50 different runs for each mutation rate value. Length of runs was 100,000 generations for the highest mutation rate (R=R_0_) and 500,000 generations for the other 3 rates. Fitness values reached within this time were very close to the optimum (within 10^-8^-10^-16^ from the optimum for the highest mutation rate and within 10^-6^-10^-10^ for the lowest mutation rate).

**population size**

Population size also showed different effects on the two phases of fitness trajectories, in similarity to mutation size and rate. The larger is the population, the slower is the fitness convergence at the first phase. The second phase was highly variable and at different repeats of the simulation different population sizes reached the best fitness (Fig. 7).

Bow-tie width showed a weak dependence on population size. In the smallest population size tested (N=50) the bow-tie obtained was not as narrow as the goal rank allows. However, between the other 3 population size values tested (100, 250, 500) there only little difference (Fig. 8).

Fig. 7: Population size affects the speed of fitness convergence at the initial phase – the larger is the population size, the faster the convergence. The finite fitness value reached is highly variable, and at different repeats of the simulation different population sizes reach the best fitness. Here we show examples of fitness temporal trajectories with different values of population size.

Fig. 8: The larger is the population size, the narrower is the waist layer (for equal number of generations). Simulation results showing layer width statistics for different population size values, where all other parameters remain intact. Statistics is based on 250 different runs for each population size value. Number of generations was 100,000 at each run.

# The emergence of bow-tie is insensitive to the internal goal structure (as long as the rank remains intact)

We compared the network structure obtained with different goal matrices that have the same rank but different internal structure. In particular we were interested whether the number of zeroes can affect the structure obtained. We found that the steepness of active node decline towards the waist can be affected. However the width and location of the waist layer are insensitive to the exact number of zeroes in the goal - See Fig. 9 for illustration. All matrices had rank 4. Matrices used as goals were (subscript denotes the number of zero terms in the matrix):

$\text{ }\mathbf{G}_{8}\text{= }\left[\begin{matrix} \text{10 10 10 10 10 10 10 0} \\ \text{10 10 10 10 10 10 10 0} \\ \text{10 10 10 10 10 10 0 10} \\ \text{10 10 10 10 10 10 0 10} \\ \text{10 10 10 10 10 0 10 10} \\ \text{10 10 10 10 10 0 10 10} \\ \text{10 10 10 10 0 10 10 10} \\ \text{10 10 10 10 0 10 10 10} \end{matrix}\right]$, $\text{ }\mathbf{G}_{16}\text{= }\left[\begin{matrix} \text{ 0 0 10 10 10 10 10 10} \\ \text{ 0 0 10 10 10 10 10 10} \\ \text{10 10 0 0 10 10 10 10} \\ \text{10 10 0 0 10 10 10 10} \\ \text{10 10 10 10 0 0 10 10} \\ \text{10 10 10 10 0 0 10 10} \\ \text{10 10 10 10 10 10 0 0} \\ \text{10 10 10 10 10 10 0 0} \end{matrix}\right]$,

$\text{ }\mathbf{G}_{32}\text{= }\left[\begin{matrix} \text{10 0 10 0 10 0 10 0} \\ \text{10 0 10 0 10 0 10 0} \\ \text{10 10 0 0 10 10 0 0} \\ \text{10 10 0 0 10 10 0 0} \\ \text{10 10 10 10 0 0 0 0} \\ \text{10 10 10 10 0 0 0 0} \\ \text{ 0 10 0 10 0 10 0 10} \\ \text{ 0 10 0 10 0 10 0 10} \end{matrix}\right]$, $\text{ }\mathbf{G}_{48}\text{= }\left[\begin{matrix} \text{ 10 10 0 0 0 0 0 0} \\ \text{ 10 10 0 0 0 0 0 0} \\ \text{ 0 0 10 10 0 0 0 0} \\ \text{ 0 0 10 10 0 0 0 0} \\ \text{ 0 0 0 0 10 10 0 0} \\ \text{ 0 0 0 0 10 10 0 0} \\ \text{ 0 0 0 0 0 0 10 10} \\ \text{ 0 0 0 0 0 0 10 10} \end{matrix}\right]$.

Fig. 9: Layer width is insensitive to the internal goal structure, as long as the rank does not change. Simulation results showing layer width statistics for goals that all have rank 4, but different number of zero terms, where all other parameters remain intact. Statistics is based on 500 different runs for each goal structure. Only runs that converged to fitness value within 0.01 from the optimum were considered (# runs analyzed = {488 498 390 497}, correspondingly). Simulation parameters: D=6, L=8. Simulations were run for 100,000 generations.

# Sum-mutations are less likely to lead to narrow bow-tie structures compared to product-mutations

We show for control the results of a similar evolutionary simulation in which sum-mutations were used instead of product mutations (and all other simulation parameters remain intact). The sum-rule is the commonly used addition of a normally distributed random number to a randomly chosen element of the matrices, which represents a mutation in the intensity of a single interaction between network components, $A_{ij}\to A_{ij}+N(0,\sigma)$.

In contrast, under product-rule mutations, an element of the matrix is multiplied by a random number drawn from a normal distribution with mean 1: $A_{ij}\to A_{ij}\cdot N(1,\sigma)$. For a comprehensive comparison of sum and product mutation rules the reader is referred to an earlier work (1). Here we compare the distributions of connection intensities obtained under these two mutational schemes in our problem (Fig. 10). Product-mutations result in a distribution that has broader tails towards both high and low-values, which is in line with the observation that more connections vanish and more nodes become inactive under product-mutations.

We analyze the number of nodes per network layer over repeats of the simulation. Ranks of the goals tested were 1 and 2. While under product-mutations a waist of width 1 evolved in the middle layer in most runs, here under sum-mutations its width was typically 3-4 (see Fig. 11 and Table 1 below for summary of simulation results).

Similarly, in the nonlinear retinal problem network structure obtained under sum-mutations did not have as narrow a waist as with product-mutations (see Fig. 12 for statistics of network structures under the two mutational schemes).

Fig. 10: Sum mutation connection intensities exhibit a narrower distribution compared to product mutations – note that the x axis is the logarithm of the absolute value of connection intensities. Histograms were produced by taking all matrix terms from 100 independent runs for each mutation type. Parameters: D=6, L=4, which results in 14,400 terms in each case. Red line designates the log of the median for each distribution.

Fig. 11: Sum-rule mutations do not lead to a narrow bow-tie for rank-deficient goals, as product-mutations do. We show here layer width statistics for goals of rank 1 (left column) and 2 (right column). Statistics is based on 360 repeats for each goal. Simulation was run for 100,000 generations each time. All runs converged to fitness value within 0.01 from the optimum.

Fig. 12: In the nonlinear problem too, sum-mutations are less likely to lead to a narrow bow-tie under rank-deficient goal compared to product mutations. Here we show a histogram of possible network structures obtained in 500 repeated runs in the nonlinear problem with either sum or product-mutations.

# Fraction of runs that did not converge to a bow-tie with narrow layer that equals the goal rank

| **Linear problem - $D=6$; $L=4$, product mutations,** $\sigma=0.1$ | | |
| --- | --- | --- |
| rank | number of runs (*) | Fraction of runs with bow-tie > rank |
| 1 | 2996 | 0.19 |
| 2 | 2986 | 0.20 |
| 3 | 1500 | 0.26 |
| 6 | 199 | 0 (**) |
|  |  |  |
| **Linear problem - $D=6$; $L=4$, product mutations,** $\sigma=0.5$(2% sign change) | | |
| rank |  |  |
| 1 | 425 | 0.37 |
| 2 | 425 | 0.48 |
| 3 | 425 | 0.51 |
|  |  |  |
| **Linear problem - $D=6$; $L=4$, sum mutations** | | |
| rank | number of runs(*) | Fraction of runs with bow-tie > rank |
| 1 | 1498 | 0.94 |
| 2 | 1484 | 0.97 |
| **Nonlinear retina problem - $D=4$; $L=3$, product mutations** | | |
| 2 | 505 | 0.16 |
| **Nonlinear retina problem - $D=4$; $L=3$, sum mutations** | | |
| 2 | 544 | 0.50 |

Table 1: Fraction of runs that did not reach a bow-tie configuration with a layer as narrow as the goal rank under either product or sum mutations for both linear and nonlinear models.

(*) - Runs were considered in the analysis only if they reached within 0.01 (or less) than the optimal fitness possible.

(**) - In the case of rank equal to 6 all network configurations must have 6 nodes at each row. Since this is also the dimension of matrices, it is not possible to have a number of nodes which is larger than the rank.

# A bow-tie evolves even if the product-mutations can change interaction sign

We tested the evolutionary simulation with product mutations that were drawn from a broader normal distribution N(1,0.5) that has probability of 2% to change the sign of the mutated element. Here too bow-ties evolved. For detailed statistics of simulation results with goals having ranks 1, 2, 3 see Fig. 13 below.

Fig 13: Bow-ties evolve even when the mutations have a significant probability to change the interaction sign. Here we drew product-mutations from a broader distribution N(1,0.5), from which 2% of the mutations would change the sign of the mutated element. We show here layer width statistics for goals of ranks 1, 2 and 3. Other simulation parameters are intact. Specifically the per-term mutation rate is 0.0014. Statistics is based on 425 independent runs for each rank. Runs were stopped when the fitness reached within 0.01 of the optimum. All runs reached this limit.

# Bow-tie dependence on noise level added to the goal

We tested the ability of the evolutionary mechanism to filter out noise added to the goal, by varying the noise level. In Fig. 14 we illustrate the width of the bow-tie obtained vs. the noise level added to a goal whose “clean rank” was 1. See noise level definition in the Methods Section (main text).

The noise was drawn from normal distributions with different standard deviation values (0.1, 0.2, 0.5, 1). Noise terms were drawn independently for each repeat of the simulation, such that the evolutionary goal was slightly different for each run (but didn’t change during the course of the run). Results are based on 780 independent runs for each noise level.

The “clean goal” was: $\mathbf{G}_{\text{clean}}=\left[\begin{matrix} \begin{matrix} 10 & 10 & 10 \\ 10 & 10 & 10 \\ 10 & 10 & 10 \end{matrix} & \begin{matrix} 10 & 10 & 10 \\ 10 & 10 & 10 \\ 10 & 10 & 10 \end{matrix} \\ \begin{matrix} 10 & 10 & 10 \\ 10 & 10 & 10 \\ 10 & 10 & 10 \end{matrix} & \begin{matrix} 10 & 10 & 10 \\ 10 & 10 & 10 \\ 10 & 10 & 10 \end{matrix} \end{matrix}\right]$.

An example of a noisy goal (lowest noise level): $\mathbf{G}_{\text{noisy}}=\left[\begin{matrix} \text{ 9.9590 10.1611 10.0919 10.0580 9.8794 10.1750} \\ \text{ 9.9630 9.9266 10.0948 10.0780 10.0377 9.8305} \\ \text{ 9.9102 9.9060 10.0166 10.0859 9.8447 10.0698} \\ \text{ 9.9437 10.0309 9.9075 9.8582 9.8845 9.9820} \\ \text{ 9.8180 10.1331 9.8766 10.0667 9.8966 10.0801} \\ \text{ 10.0806 10.0267 10.0364 10.0566 9.9908 10.2014} \end{matrix}\right]$.

Figs. 14-15 illustrate the effect of noise added to the goal on the number of active nodes per layer.

Fig. 14: Bow-tie width increases with noise level added to the goal. The evolutionary mechanism is able to filter out noise to a certain extent, and expose the clean rank of the goal, however when noise becomes too large the evolutionary mechanism fails in filtering. Here the clean rank of the goal was 1 and the noise was drawn from normal distributions with increasing standard deviations. Noise level was defined as the absolute value of the difference between noisy and clean goal norms divided by the clean goal norm (see Methods Section).

Fig. 15: Median number of active nodes per layer for varying noise levels added to the goal. Results refer to the same simulations as in the previous figure.

# Product-mutations can filter temporal noise efficiently and lead to bow-tie; sum-mutations cannot.

Whereas in the previous scenario the goal rank was noisy, but the goal itself remained constant throughout every simulation run, temporal fluctuations are also ubiquitous in biological networks. To test the effect of temporal fluctuations, we added statistically independent noise realizations (white noise) to all matrix entries (and also to the goal) at each fitness evaluation, namely: $F=-||(\mathbf{A}^{(L)}+\mathbf{\varepsilon}_{L})(\mathbf{A}^{(L-1)}+\mathbf{\varepsilon}_{L-1})\ldots(\mathbf{A}^{(1)}+\mathbf{\varepsilon}_{1})-(\mathbf{G}+\mathbf{\varepsilon}_{G})||$, where $\mathbf{\varepsilon}_{i}$are independent noise realizations drawn from a Gaussian distribution $N(0,\sigma)$ with different values of $\sigma$ varying between 0.001 to 0.2. The goal had rank 1. We estimated the effect of temporal fluctuations on evolution through the temporal fluctuations in fitness values, calculated over the last 5000 generations in the simulation, when the run has already converged (runs were for either 50,000 or 100,000 generations each). We used the same goal as in the previous section. As this noise changes in a higher frequency than the typical evolutionary timescale (the mutation rate), we expected that the system will be able to filter it out to some extent. We compared the ability of evolution by means of either product or sum mutations to cope with this temporal noise. We find that product-mutations filter out the noise much more efficiently than sum-mutations. When the clean goal rank was 1, the network structure evolved by product-rule evolutionary scheme was unaffected until the relative effect of these fluctuations on fitness reached values of 0.3. Sum mutations, in contrast, led to bow-tie of width 3 even in the absence of noise and bow-tie width sharply increased to 5 when temporal noise was added. See Fig. 16 for illustration.

Fig. 16: Product mutations can filter out temporal noise efficiently and lead to bow-tie structures, while sum-mutations cannot. Evolutionary simulations were repeated with either sum or product mutation scheme. Bow-tie width was calculated for either mutational scheme. Results are the median over 150 independent repeats. Temporal fitness fluctuations were calculated over the last 5000 generations of the run, when fitness mean has already stabilized. These temporal fitness fluctuations are then used as a noise measure. We plot the number of active nodes vs. 3 possible variables (the x-axis): std of noise added in simulation, std (or std/mean) of the resultant fitness fluctuations. Simulations were run for either 50,000 (3 lowest noise levels) or 100,000 (higher noise levels) generations. As a control, we calculated the number of active nodes in the network under various noise realizations (having the same statistics) and found it insensitive.

# Estimation of noise in a biological network

In the main text and in the previous section we present simulation results when noise was added to the network. To assess the noise level in a real biological network, we estimate in this section the magnitude of noise in a network due to spurious gene expression. Despite the large body of work on this topic, results mostly refer to variability at the single gene level, while measurements and theoretical works on noise at the network level are scarce (2–5). The main hurdle is how exactly noise is propagated between network elements.

Specifically, noise in linear cascades was modeled by Thattai and van Oudenaarden (3) and Levine and Hwa (5). This is basically a special case of the model presented here – a multi-layered network with only a single node at each layer.

To remind the reader, the noise scheme used in our simulations is:

$$\text{Output = }\left(\mathbf{A}^{(L)}+\mathbf{\varepsilon}^{(L)}\right)\left(\mathbf{A}^{(L-1)}+\mathbf{\varepsilon}^{(L-1)}\right)...\left(\mathbf{A}^{(1)}+\mathbf{\varepsilon}^{(1)}\right)\text{Input}$$

Here noise is injected in each layer in a multiplicative manner. $\mathbf{\varepsilon}^{(i)}$ is a matrix with the same dimensionality as the $A$ matrices. The rationale for this difference lies in the source of the noise considered here. The noise injected in each layer is assumed to originate from non-specific binding of regulators to targets in the next layer. Thus the affinity matrices, $\mathbf{A}$-s, are augmented by the $\mathbf{\varepsilon}^{(i)}$. Accordingly the amplitude of the noise is estimated by the affinity of a regulator to bind non-specifically to a target.

In order to quantify the relative contribution of noise in network models, we estimate below spurious gene expression due to non-specific binding of RNA polymerase to DNA in bacteria. RNA Polymerase can bind to random DNA sequences, some of which might be genes. Thus estimation of non-specific binding provides an upper bound to spurious gene expression. In Bintu et.al 2005 (8), an order of magnitude estimate for the ratio of specific to non-specific binding is given. It is based on the probability of binding of RNAP to promoter as a function of the number of RNAP molecules for two different promoters. The assumption is that the DNA molecule serves as a reservoir for the RNAP molecules. Hence the number of non-specific sites is of the order of 10^6^ (for E.Coli). The binding energy difference is calculated using the simple relation $\Delta E=k_{B}T\ln(K_{s}/K_{ns})$, where the equilibrium dissociation constants for specific binding (K_s_) and non-specific binding (K_ns_) are taken from in vitro measurements. In particular, making the simplest assumption that the genomic background for RNAP is given only by the non-specific binding of RNAP with DNA, K_ns_ is taken to be 10 000 nM , and K_s_ is taken to be between 10-500 nM. This gives a noise estimate of 0.001-0.05. The corresponding $\Delta E$ is in the order of 3-8 k_B_T

In comparison, typical numbers used in the simulation are:

$L=4$ (number of connection matrices), $D=6$ (number of nodes per layer) and $g=10$ (a term in the goal matrix). At one extreme, if all network connections are equal (the opposite of bow-tie), each matrix entry then equals:

$$a=\left(\frac{g}{D^{L-1}}\right)^{1/L}=0.46$$

In simulations testing temporal noise we added i.i.d noise realizations to each matrix entry at every generation. The noise terms were drawn from a normal distribution with mean 0 and std in the range 0.001-0.2.

Assuming uniform weights across the network the relative weight of the noise (non-specific/specific) is in the range 0.002-0.43.

In the other extreme, if the network evolved to satisfy a goal of rank 1 and is the most parsimonious possible, it has $D$ nodes only at the input and output layers and only 1 node at all other layers. Assuming all active nodes have equal weights, then a single node has weight:

$$b=\left(g\right)^{1/L}=1.8$$

Injecting similar noise levels then amounts to relative noise in the range 0.0005-0.1.

To conclude, the noise levels we used in these simulations cover well the fluctuation range expected due to non-specific binding in gene regulatory networks.

# Change in network size – bow-tie is typically ossified

In all previous simulations we started with a network whose elements are all very weakly interacting and then let all interactions evolve simultaneously to satisfy the goal. A more biologically realistic scenario however is a network that evolves gradually, where at each step additional element(s) add up to an already functional network which performs some task. To examine this situation we initially simulated a low-dimensional network that evolves to satisfy a low-dimensional goal. Then at each step the goal dimension grew by 1 and the network size grew accordingly and evolved to satisfy the extended goal. Importantly, we kept the goal rank intact (=1) and only changed its size, namely added inputs and outputs to the network.

An example of these simulation results is shown in Fig. 17, where the network dimension gradually grows from 3 to 8. Interestingly, the bow-tie is “ossified”: a bow-tie node that evolved in low dimensional network most likely remains a bow-tie even when the network expands (rather than being replaced by another node at the same layer). This effect creates a correlation between node “age” and its likelihood of becoming a bow-tie. Network growth then mainly incurs addition of peripheral nodes (inputs and outputs) rather than changes in the network core. In contrast when the whole network evolves simultaneously all intermediate layer nodes are equally likely to become a bow-tie.

Fig 17: When the network grows step-by-step, a bow-tie formed in a small network is typically ossified. Simulation results showing a network that gradually grows from $D=3$ to $D=8$. Each matrix in this figure represents the interaction intensities between adjacent network layers using a color code (white = zero, the darker – the stronger). Each column of matrices represents a single network at a specific dimension. In all dimensions smaller than 8 we padded the matrices with additional zeros to make them all 8X8 for presentation purpose. Imbalance between interaction intensities (namely a bow-tie) already forms in the smallest dimension ($D=3$). Interestingly, this core of active interactions is preserved even when the network grows. One can observe that even in the larger network the dominant interaction (dark pixels) are at the most ancient part of the network, which is at the upper left corner of the matrix. Each step of the simulation was run for 50,000 generations so that the network reached high fitness values for that specific goal (typically ~10^-6^). Simulation parameters: mutation size = 0.1.

**References**

1. Friedlander T, Mayo AE, Tlusty T, Alon U (2013) Mutation Rules and the Evolution of Sparseness and Modularity in Biological Systems. *PLoS One* 8(8). Available at: http://www.ncbi.nlm.nih.gov/pmc/articles/PMC3735639/.

2. Arbel-Goren R, et al. (2013) Effects of post-transcriptional regulation on phenotypic noise in Escherichia coli. *Nucleic acids research* 41(9):4825–4834.

3. Thattai M, van Oudenaarden A (2002) Attenuation of noise in ultrasensitive signaling cascades. *Biophysical journal* 82(6):2943–2950.

4. Osella M, Bosia C, Corá D, Caselle M (2011) The role of incoherent microRNA-mediated feedforward loops in noise buffering. *PLoS computational biology* 7(3):e1001101.

5. Levine E, Hwa T (2007) Stochastic fluctuations in metabolic pathways. *PNAS* 104(22):9224–9229.

6. Gerland U, Moroz JD, Hwa T (2002) Physical constraints and functional characteristics of transcription factor–DNA interaction. *Proceedings of the National Academy of Sciences* 99(19):12015–12020.

7. Wunderlich Z, Mirny LA (2009) Different gene regulation strategies revealed by analysis of binding motifs. *Trends in Genetics* 25(10):434–440.

8. Bintu L, et al. (2005) Transcriptional regulation by the numbers: models. *Current Opinion in Genetics & Development* 15(2):116–124.
